# Genome Comparison Reveals Inversions and Alternative Evolutionary History of Nutritional Endosymbionts in Planthoppers (Hemiptera: Fulgoromorpha)

**DOI:** 10.1093/gbe/evad120

**Published:** 2023-07-01

**Authors:** Junchen Deng, Gordon M Bennett, Diego C Franco, Monika Prus-Frankowska, Adam Stroiński, Anna Michalik, Piotr Łukasik

**Affiliations:** Institute of Environmental Sciences, Faculty of Biology, Jagiellonian University, Kraków, Poland; Doctoral School of Exact and Natural Sciences, Jagiellonian University, Kraków, Poland; Department of Life and Environmental Sciences, University of California, Merced, California, USA; Institute of Environmental Sciences, Faculty of Biology, Jagiellonian University, Kraków, Poland; Institute of Environmental Sciences, Faculty of Biology, Jagiellonian University, Kraków, Poland; Polish Academy of Sciences, Museum and Institute of Zoology, Warsaw, Poland; Department of Developmental Biology and Morphology of Invertebrates, Institute of Zoology and Biomedical Research, Faculty of Biology, Jagiellonian University, Kraków, Poland; Institute of Environmental Sciences, Faculty of Biology, Jagiellonian University, Kraków, Poland

**Keywords:** endosymbiosis, planthopper, betaproteobacteria, genome inversion, diversity and origin

## Abstract

The evolutionary success of sap-feeding hemipteran insects in the suborder Auchenorrhyncha was enabled by nutritional contributions from their heritable endosymbiotic bacteria. However, the symbiont diversity, functions, and evolutionary origins in this large insect group have not been broadly characterized using genomic tools. In particular, the origins and relationships among ancient betaproteobacterial symbionts *Vidania* (in Fulgoromorpha) and *Nasuia*/*Zinderia* (in Cicadomorpha) are uncertain. Here, we characterized the genomes of *Vidania* and *Sulcia* from three *Pyrops* planthoppers (family Fulgoridae) to understand their metabolic functions and evolutionary histories. We find that, like in previously characterized planthoppers, these symbionts share nutritional responsibilities, with *Vidania* providing seven out of ten essential amino acids. *Sulcia* lineages across the Auchenorrhyncha have a highly conserved genome but with multiple independent rearrangements occurring in an early ancestor of Cicadomorpha or Fulgoromorpha and in a few succeeding lineages. Genomic synteny was also observed within each of the betaproteobacterial symbiont genera *Nasuia*, *Zinderia,* and *Vidania*, but not across them, which challenges the expectation of a shared ancestry for these symbionts. The further comparison of other biological traits strongly suggests an independent origin of *Vidania* early in the planthopper evolution and possibly of *Nasuia* and *Zinderia* in their respective host lineages. This hypothesis further links the potential acquisition of novel nutritional endosymbiont lineages with the emergence of auchenorrhynchan superfamilies.

**Significance**

The reconstruction of the evolutionary history of extremely reduced and fast-evolving ancient bacterial symbionts often brings challenges to conventional approaches. *Sulcia* and betaproteobacterial symbionts (*Vidania*, *Nasuia*, and *Zinderia*) are such symbionts that have co-evolved with Auchenorrhyncha insects for millions of years. A single origin of *Vidania*, *Nasuia*, and *Zinderia* was widely assumed, despite the lack of convincing evidence from genomics and phylogenetics in the only study that attempted to compare them so far. Here, we revisit this hypothesis and propose an independent origin of each betaproteobacterial symbiont by showing their global lack of synteny contrasting to the conserved gene order observed in *Sulcia*. Our work indicates the limit of phylogenetic tools and highlights the importance of a holistic biological approach in solving the origin and history of ancient bacterial symbionts.

## Introduction

Symbiosis, defined as the long-term interaction between different organisms living closely together (McCutcheon 2021), plays a critical role in the evolution of life on Earth. The most intimate are endosymbioses where one of the partners lives within the cells of another. The best-known example is the endosymbiosis between an alphaproteobacterium and an archaeon that originated around two billion years ago, where the former partner evolved into what we now know as the mitochondrion within a eukaryotic cell (Imachi et al. 2020). Among the different types of eukaryote-bacterial endosymbiosis that evolved subsequently, obligate nutritional endosymbioses have emerged as fundamental drivers of animal biodiversity, particularly in insects (McCutcheon et al. 2019). Like mitochondria, obligate endosymbionts undergo strict vertical transmission from mother to offspring and depend on their hosts for many essential cellular functions. Although some of these symbiotic relationships seem to have started relatively recently (Husnik and McCutcheon 2016; Michalik et al. 2021), others have lasted for tens or even hundreds of millions of years (Baumann 2005; Bennett and Moran 2015).

A common feature of these ancient relationships is the extremely small genome of the microbial symbiont. The genomes of the beneficial endosymbionts generally undergo rapid and substantial gene loss due to the relaxation of selection on redundant and nonessential genes, elevated mutation rates, and strong genetic drift resulting from population bottlenecks during the vertical transmission (McCutcheon et al. 2019). In extreme cases, genome size becomes so small, barely over 100 kilobases, that only functionally essential genes remain, including those involved in transcription, translation, and nutritional provisioning (Bennett and Moran 2013). As these evolutionary processes proceed, genetic drift drives the fixation of deleterious mutations even in these essential genes, which can be lost over time in these tiny genomes (Vasquez and Bennett 2022). Nevertheless, it is typically observed that the genomes of mutualistic endosymbionts establish a high level of synteny fairly early in their evolution (Patiño-Navarrete et al. 2013; Williams and Wernegreen 2015; Chong et al. 2019) with some caveats (e.g., Sloan and Moran 2013). Strict vertical transmission and lack of an environmental phase eliminate the opportunity for recombination between symbiont species and strains (Perreau and Moran 2021). In addition, the lack of transposable elements and the loss of genes involved in DNA recombination during the extreme size shrinkage also makes structural changes less likely to happen (Silva et al. 2003; Chong et al. 2019).

Among the best-known nutritional endosymbioses are those in the sap-feeding insect clade Auchenorrhyncha (Hemiptera), which comprises cicadas, spittlebugs, planthoppers, leafhoppers, and treehoppers. To overcome the poor nutritional quality of phloem and xylem plant saps, Auchenorrhyncha species depend on intracellular bacterial symbionts for essential amino acids (EAAs) and vitamins. One of these symbionts, “*Candidatus* Sulcia muelleri” (Bacteroidetes; hereafter: *Sulcia*) established in the common ancestors of Auchenorrhyncha ∼300 million years (Myr) ago (Moran et al. 2005; Bennett and Moran 2013; Johnson et al. 2018). Along with *Sulcia*, a diversity of coresident bacterial symbionts have been described, including Betaproteobacteria “*Ca*. Vidania fulgoroideae” (*Vidania*) in planthoppers, “*Ca.* Nasuia deltocephalinicola” (*Nasuia*) in treehoppers and leafhoppers, “*Ca*. Zinderia insecticola” (*Zinderia*) in spittlebugs, and an alphaproteobacterium “*Ca.* Hodgkinia cicadicola” (*Hodgkinia*) in cicadas (McCutcheon and Moran 2010; Bennett and Moran 2013). *Sulcia* and these coresident symbionts together provide a complete set of EAAs for the hosts. Among these cosymbionts, the betaproteobacteria *Vidania*, *Zinderia*, and *Nasuia* (referred to as beta-symbionts) share a few genomic characteristics (e.g., extremely reduced genomes) and phylogenomic reconstructions suggested that they might have descended from a single ancestral betaproteobacterial lineage (Bennett and Moran 2013; Koga et al. 2013; Bennett and Mao 2018), which was replaced by the alphaproteobacterial symbiont *Hodgkinia* in the ancestor of cicadas. However, the genome dissimilarity among beta-symbionts and several known problems with phylogenetic reconstructions, such as the extremely reduced genome and the long-branch attraction, make the hypothesis speculative (discussed in Urban and Cryan 2012; Bennett and Mao 2018).

Among all Auchenorrhyncha clades, the least-understood is the planthoppers (infraorder Fulgoromorpha) and their endosymbionts. Planthoppers are an ecologically diverse group of >12,000 described species grouped into over 20 families; many of them are economically significant as agricultural pests (Urban and Cryan 2007; Dietrich 2009). The early evidence of endosymbionts in planthoppers was revealed by the extensive microscopy work of Müller and Buchner, who described the common presence of two endosymbionts, “a-symbiont” and “x-symbiont”, often accompanied by additional bacteria or fungi (Müller 1940, 1949; Buchner 1965). These two common symbionts were identified over half a century later as *Sulcia* and *Vidania*, respectively, with the help of broad sampling and molecular markers (Moran et al. 2005; Gonella et al. 2011; Urban and Cryan 2012). However, the genomic study of endosymbionts in Fulgoromorpha was a completely white page until 2018 when the first complete genomes of *Sulcia* and *Vidania* from a Hawaiian planthopper *Oliarus filicicola* (family Cixiidae) were published (Bennett and Mao 2018). They reported unique features in these planthopper symbionts compared to symbionts in the other Auchenorrhyncha infraorder (Cicadomorpha), including a large chromosomal inversion in *Sulcia,* a dominant nutritional role of *Vidania* over *Sulcia*, and several genomic differences in *Vidania* compared to beta-symbionts in Cicadomorpha, such as the retention of UGA stop codon and the usage of a different methionine biosynthesis pathway. Recent studies confirmed these patterns in two *Oliarus* planthoppers (Gossett et al. 2023) and other three species from a divergent family Dictyopharidae (Michalik et al. 2021), indicating a likely different evolutionary trajectory of endosymbiosis in Fulgoromorpha than in Cicadomorpha. However, with the symbiont genome-level data available for only five species representing two out of over 20 planthopper families, our comprehension of the evolutionary history of this insect group is far from complete.

To better understand the endosymbiosis in Fulgoromorpha, we characterized *Sulcia* and *Vidania* genomes from three planthopper species in the genus *Pyrops* (family Fulgoridae). This genus includes some of the largest and most spectacular planthopper species, often characterized by elongated head and contrasting color patterns on the wings (Urban and Cryan 2009). We asked 1) how similar *Sulcia* and *Vidania* are to previously published planthopper symbiont strains, using whole genome assembly from high-throughput sequencing data, and comparative genomics approaches. After discovering a novel rearrangement in *Sulcia* genomes from *Pyrops*, we then asked 2) how common genome rearrangements have been in *Sulcia*, and 3) whether genome organization and function across *Vidania*, *Nasuia*, and *Zinderia* support hypotheses about their shared origin. We show that *Sulcia* and *Vidania* from *Pyrops* planthoppers are highly similar to previously sequenced strains in other planthoppers. *Sulcia* lineages from both Fulgoromorpha and Cicadomorpha retain a conserved gene order, despite having gone through genome rearrangements in some host clades. In contrast, the unexpected lack of synteny between beta-symbiont genomes strongly argues against their shared ancestry. Although our improved phylogeny still placed *Vidania*, *Nasuia*, and *Zinderia* within a highly supported monophyletic group, the lingering issue of long-branch attraction in these extremely reduced genomes makes the interpretation from phylogeny inconclusive. Taken together, our data favored multiple origins of beta-symbionts, particularly for *Vidania*.

## Results

### Each *Pyrops* Planthopper Harbors Four Types of Bacteriome-associated Endosymbionts

We obtained 11.2, 9.2, and 8.0 Gb of 2 × 150 bp Illumina data, in addition to 2.5, 1.5, and 1.5 Gb of Nanopore data (N50 = 4.9, 3.2, and 4.7 kb), for three *Pyrops* species (*P. lathburii*, *P. clavatus*, and *P. viridirostris*), respectively. The output of phyloFlash and anvi’o platform confirmed the presence of four distinct endosymbionts in each *Pyrops* species. In each host, we identified the two ancient symbionts, *Sulcia* and *Vidania*, along with two gammaproteobacterial symbionts. These results were confirmed by metagenomic assemblies. The assemblies using short and long reads resulted in complete circular genomes of *Sulcia* and *Vidania* and fragmented genomes of the gammaproteobacterial symbionts.

**Fig. 1:**
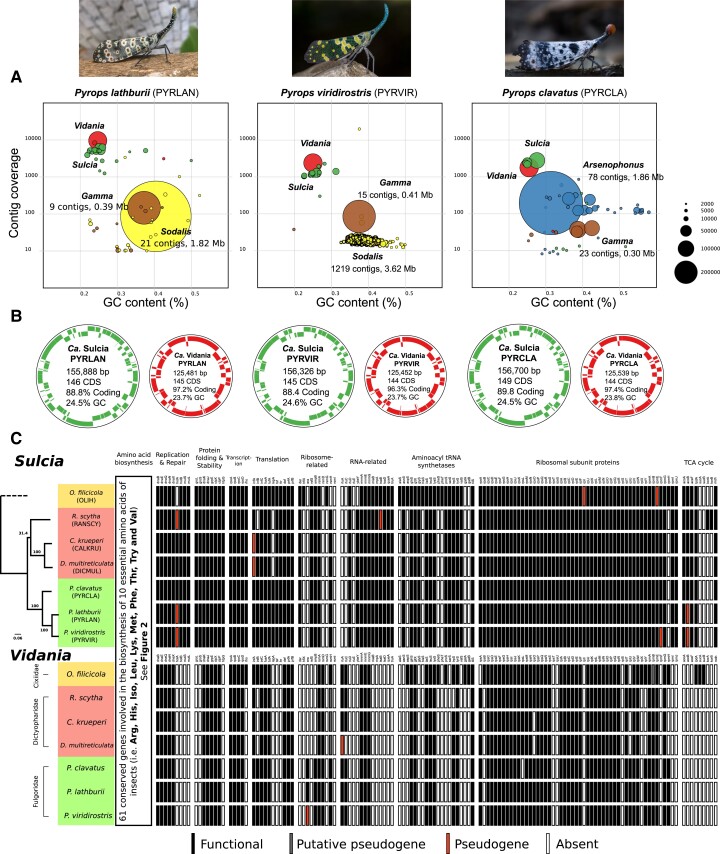
The summary of metagenomic assemblies of three *Pyrops* planthoppers. (*A*) The visual representation of endosymbiont diversity based on short-read assembly. Each circle represents a single contig. The circle size is proportional to the length of the contig as it is plotted in GC% and read coverage space. Different colors are applied to different endosymbionts: *Sulcia* (green), *Vidania* (red), *Sodalis* (yellow), *Arsenophonus* (blue), and *Gamma* (brown). Note that *Sulcia* genomes were fragmented in the short-read assemblies but were circular in long-read assemblies, which were then polished by short reads to achieve the final quality. (*B*) The visualization of the circular genomes of *Sulcia* and *Vidania*. Genes on forward and reverse strands are shown in colors. Extra information on the genomes, including length (bp), the number of coding DNA sequences (CDS), coding density, and GC content, is shown inside each genome circle. (*C*) The comparison of gene set between *Sulcia* and *Vidania* lineages from seven planthopper species representing three families. Each bar represents a single gene with a name abbreviation on the top. Genes are classified into functional (black), putative pseudogene (dark gray), pseudogene (red), and absence (white). The maximum likelihood tree of seven host species based on the concatenated ten mitochondrial markers (*nad2*, *cox1*, *cox2*, *atp6*, *cox3*, *nad3*, *nad6*, *cob*, *nad1*, and *rrnL*) is shown on the left. The dotted branch indicates the position of a related species described in the methods section. Insect photographs by Luan Mai Sy, Dr. Vijay Anand Ismavel, and Supratim Deb, originally published on iNaturalist.org.

*Sulcia* and *Vidania* genome sizes range from 155.8 to 156.7 kb and 125.4 to 125.6 kb, respectively (fig. 1*B*). These sizes are comparable to genomes in family Dictyopharidae (142–148 kb for *Sulcia* and 122–125 kb for *Vidania*) and Cixiidae (157 kb for *Sulcia* and 136 kb for *Vidania*) (Bennett and Mao 2018; Michalik et al. 2021). The Gammaproteobacteria have larger, more complex genomes. All three hosts have a gammaproteobacterial symbiont (denoted as Gamma in fig. 1*A*) with relatively small genomes (292–414 kb; 9–23 contigs). The top “blastn” hits for 16S rRNA sequences against the NCBI database indicate that this symbiont is most closely related to the secondary symbionts of mealybugs and psyllids (90–92% similarity; Sloan and Moran 2012; Szabó et al. 2017) within the *Sodalis* clade. In addition, *Pyrops lathburii* and *P. viridirostris* have *Sodalis* symbionts (96% 16S rRNA sequence similarity to the free-living strain *Sodalis* praecaptivus) with genomes of 1.82–3.62 Mb (21–1219 contigs) while *P. clavatus* has an *Arsenophonus* with a genome of 1.86 Mb (78 contigs).

### The Conservation of *Sulcia* and *Vidania* Genome Contents in *Pyrops* Planthoppers

**Fig. 2:**
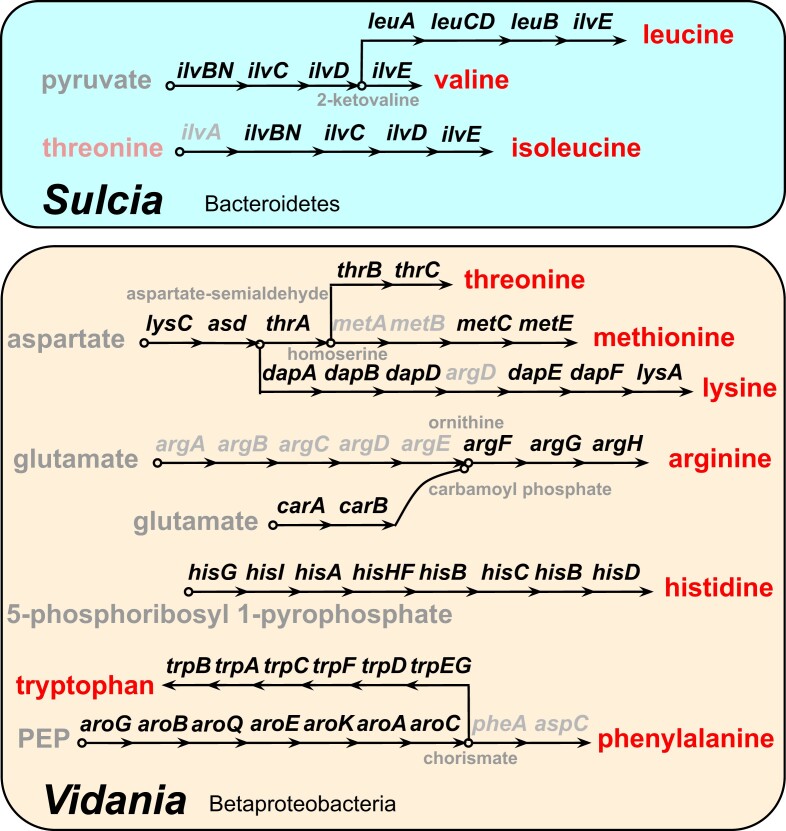
The reconstruction of biosynthesis pathways of ten EAAs in *Sulcia* and *Vidania* from three *Pyrops* planthoppers. Each arrow represents a single step in the reaction, with the abbreviated name of the gene shown above. Genes missing from the pathway are shown in gray. EAAs are in red. Chemical compounds involved in some steps are also shown.

The genomes of both *Sulcia* and *Vidania* are highly reduced and gene-dense. Each *Sulcia* genome contains 145–149 predicted protein-coding sequences (CDSs), 29 tRNAs, and a complete ribosomal operon (fig. 1*B*). Similarly, each *Vidania* genome contains 144–145 CDSs, 24–25 tRNAs, and a complete ribosomal operon (fig. 1*B*). Regarding the symbionts’ nutritional roles, *Sulcia* encodes biosynthesis pathways for three EAAs (leucine, valine, and isoleucine) while *Vidania* for seven (methionine, histidine, tryptophan, threonine, lysine, arginine, and phenylalanine) (fig. 2). Gene losses have occurred in several pathways of both *Sulcia* and *Vidania*, including in *Vidania* the last step (*pheA* and *aspC*) in phenylalanine biosynthesis pathway, the synthesis of ornithine from glutamate (*argABCDE*) in the arginine pathway, and the cysteine pathway (*metAB*) in methionine biosynthesis, and in *Sulcia* the initial step (*ilvA*) in isoleucine biosynthesis (fig. 2).

Within the genus *Pyrops*, gene content is highly similar among *Sulcia* and *Vidania*, with only a few differences. *Sulcia* in *P. clavatus* retains the ability to synthesize the subunit delta of the gamma complex of DNA polymerase III (*holA*) involved in DNA replication, as well as the subunit beta of Pyruvate dehydrogenase E1 component (*acoB*) involved in the citric acid cycle (TCA cycle). Both of these genes have been pseudogenized in the other two *Sulcia* lineages (fig. 1*C*). In *Vidania*, the gene encoding one ribosomal subunit protein (*rpsL*) was lost from *P. clavatus*.

Gene content between *Pyrops* symbionts and the closely related family Dictyopharidae (RANSCY, CALKRU, and DICMUL; fig. 1*C*) is also similar. *Sulcia* in the dictyopharids has lost the translation elongation factor 4 (*lepA*). There was an additional loss of translation initiation factor IF-2 (*infB*) in *Sulcia*-RANSCY. In contrast, *Sulcia*-RANSCY retains the ability to synthesize succinyl-CoA from 2-oxoglutarate through the pathway catalyzed by 2-oxoglutarate oxidoreductase (*korA* and *korB*), which was lost in all other known *Sulcia* lineages from planthoppers. In *Vidania*, translation initiation factor IF-2 (*infB*) was lost in three Dictyopharidae strains.

Finally, compared with symbionts from the more distantly related Cixiidae species, *Oliarus filicicola* (strain OLIH), gene content differences are more pronounced (fig. 1*C*). The most notable difference is within the gene set coding for aminoacyl-tRNA synthetases. *Sulcia* from *Pyrops* and Dictyopharidae have lost five genes for tRNA synthetase (*hisS*, *pheS*, *pheT*, *aspS*, and *alaS*) relative to *Sulcia*-OLIH. Likewise, *Vidania* from *Pyrops* and Dictyopharidae has lost five genes for tRNA synthetases (*aspS*, *gatA*, *gatB*, *pheS*, and *proS*). An additional synthetase loss (*trpS*) occurred in *Vidania*-RANSCY. Furthermore, *Vidania* occurring in *Pyrops* and Dictyopharidae have distinctly lost the complete gene set (*lpd*, *sucA*, and *sucB*) involved in converting 2-oxoglutarate into succinyl-CoA in the TCA cycle. Two genes involved in the assembly of iron-sulfur clusters (*erpA* and *iscU*, not shown in the figure) responsible for electron transfer were also lost in these two clades.

### Several Genome Rearrangements Have Happened in *Sulcia* Across Auchenorrhyncha

**Fig. 3:**
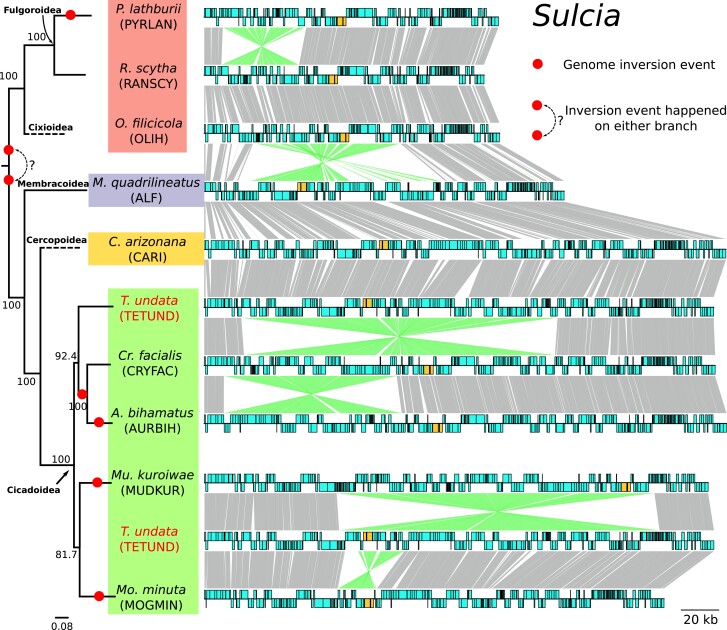
Genome comparison between *Sulcia* lineages. Each circular genome is represented linearly, starting from the same position (*lipB* gene). Genes on the forward and reverse strands are shown on each side as blue boxes. Ribosomal RNAs are colored orange. Homologous genes are connected by gray lines between genomes. Lines connecting inverted regions are colored green. The maximum likelihood tree of ten host species based on the concatenated ten mitochondrial markers (*nad2*, *cox1*, *cox2*, *atp6*, *cox3*, *nad3*, *nad6*, *cob*, *nad1*, and *rrnL*) is shown on the left. The dotted branch indicates the position of a related species, as described in the Methods. Note that the lineages shown here represent all inversions we found. Other genomes with the same organization were omitted from this figure. The comparison of all 41 *Sulcia* lineages can be found in supplementary material figure S1, Supplementary Material online.

As found previously, *Sulcia*-OLIH (Cixiidae) had a large 78 kb inversion compared to *Sulcia*-ALF from the leafhopper *Macrosteles quadrilineatus* (Bennett and Mao 2018). *Sulcia* from Dictyopharidae, which is distantly related to cixiids, were collinear to *Sulcia*-OLIH, indicating that this inversion is an ancestral feature of Fulgoromorpha. Here, we observed a novel genome rearrangement (∼43 kb including 32 CDSs), in *Sulcia* from *Pyrops* planthoppers compared to cixiid and dictyopharid *Sulcia* (fig. 3; supplementary material fig. S1, Supplementary Material online). By further investigating all 41 previously published *Sulcia* genomes across the Auchenorrhyncha, we identified four other instances of genome rearrangements. All of them have occurred in cicadas (Cicadoidea) and were confined to the genera *Auritibicen* (∼94 kb, 77 CDSs), *Cryptotympana* (∼165 kb, 133 CDSs), *Muda* (∼169 kb, 162 CDSs), and *Mogannia* (∼20 kb, 16 CDSs) (fig. 3). In contrast, all *Sulcia* lineages from spittlebugs (Cercopoidea) and leafhoppers and treehoppers (Membracoidea) were collinear with cicada *Sulcia* lineages presenting the ancestral genome organization (e.g., *Tettigades undata* TETUND) (fig. 3; supplementary material fig. S1, Supplementary Material online). Together, genomes from across the Auchenorrhyncha indicate that an ancestral rearrangement must have occurred in the common ancestor of either Fulgoromorpha or Cicadomorpha shortly after they diverged.

### Betaproteobacterial Phylogeny and the Lack of Synteny Between Beta-Symbiont Lineages

**Fig. 4:**
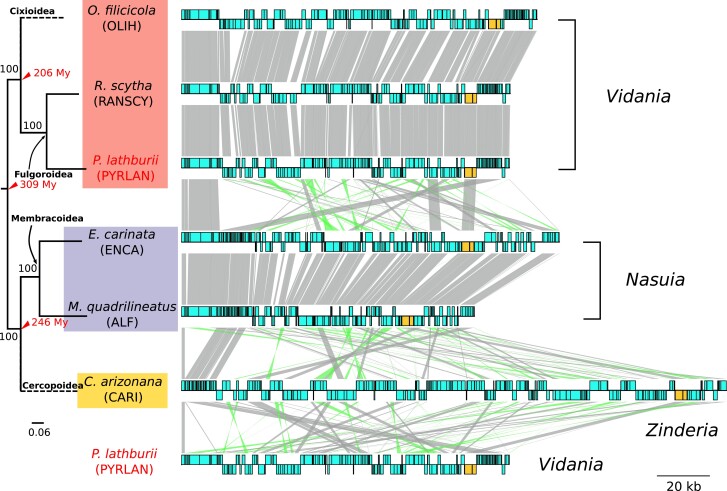
Genome comparison between beta-symbionts (*Vidania*, *Nasuia*, and *Zinderia*). Each circular genome is represented linearly, starting from the same position (*tufA* gene). Genes on the forward and reverse strands are shown on each side as blue boxes. Ribosomal RNAs are colored in orange. Homologous genes are connected by gray lines between genomes. Lines connecting inverted regions are colored in green. The maximum likelihood tree of six host species based on the concatenated ten mitochondrial markers (*nad2*, *cox1*, *cox2*, *atp6*, *cox3*, *nad3*, *nad6*, *cob*, *nad1*, and *rrnL*) is shown on the left. The dotted branch indicates the position of a related species described in the methods section. The node age is indicated according to Johnson et al. (2018).

Like *Sulcia*, *Vidania* lineages retained a shared gene order (fig. 4 and supplementary material fig. S2, Supplementary Material online). This collinearity can also be observed in *Nasuia* (fig. 4; See also Vasquez and Bennett 2022). Remarkably, however, the three major beta-symbiont lineages share very little synteny (fig. 4). The only syntenic regions across all three betaproteobacterial lineages are the more universally conserved ribosomal protein gene clusters (Barloy-Hubler et al. 2001).

**Fig. 5:**
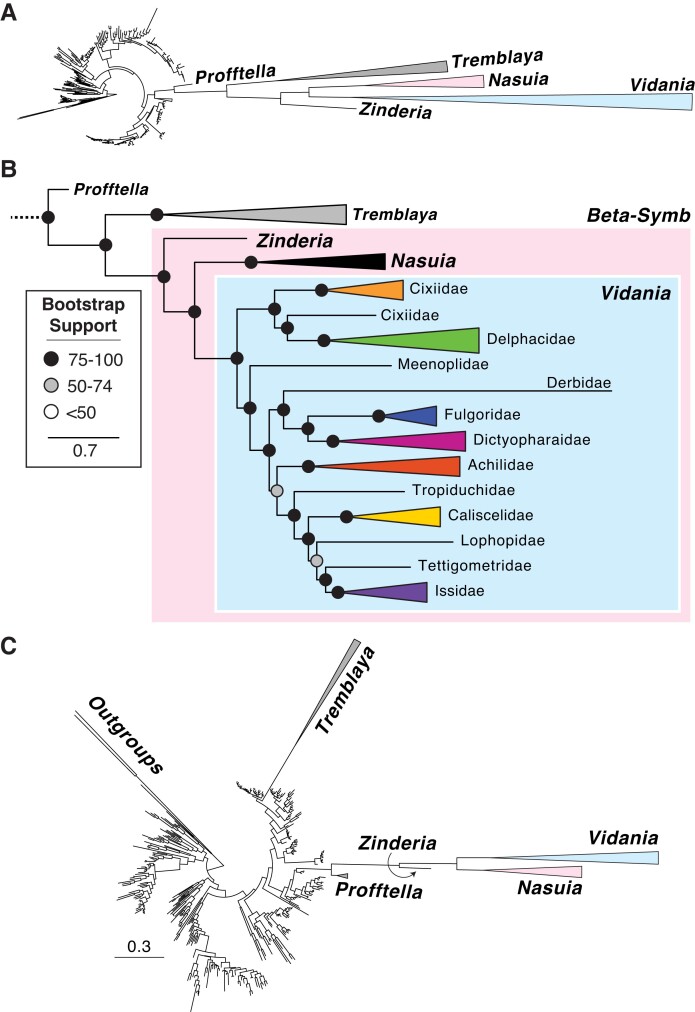
Phylogenetic relationships of the *Betaproteobacteria*. (*A*) Maximum likelihood phylogeny reconstructed with 289 taxa and 7,240 sites (31 genes). Symbiont clades are collapsed. (*B*) The zoom-in view of phylogeny shows the relationships among symbionts. *Vidania* clade is collapsed and labeled according to its host family. (*C*) Concatenated 16*S* + 23*S* rRNA Maximum likelihood phylogeny reconstructed with 382 taxa and 6,011 nucleotide sites. Symbiont clades are collapsed. The full trees can be found in the supplementary material Data S3–4 and S6, Supplementary Material online.

Results from both amino acid, Dayhoff6-recoded, and ribosomal RNA (rRNA)-based phylogenetic analyses placed the Auchenorrhyncha symbionts into a highly supported monophyletic clade (fig. 5*A*-*C*; BS = 100; supplementary material Data Files S3–4 and S6, Supplementary Material online). This clade was placed within the Oxalobacteriaceae family (Burkholderiales) as has been found several times previously (BS = 91; e.g., McCutcheon and Moran 2010; Mao et al. 2017). We note that both our phylogenetic efforts revealed long-branch attraction between symbionts of scale insects and psyllids (e.g., *Tremblaya* and *Profftella*, respectively) and the symbionts of Auchenorrhyncha, placing them in a well-supported monophyletic clade. The phylogeny based on 16S and 23S rRNAs suffered less from long-branch attraction, as it successfully split off *Tremblaya* as an independent clade (BS = 82–100), but it still groups *Profftella* with Auchenorrhyncha symbionts (BS = 100). These insects are anciently diverged, occurring in the Sternorrhyncha suborder (Hemiptera), and are unlikely to share a common symbiont. Furthermore, the placement of the spittlebug symbiont, *Zinderia* (BS = 100), basal to *Vidania* and *Nasuia*, does not match the putative relationships for these major auchenorrhynchan clades (e.g., Johnson et al. 2018; Skinner et al. 2020). In particular, spittlebugs and leafhoppers are sister groups within the Cicadomorpha; the Cicadomorpha is an early diverging sister clade of the Fulgoromorpha planthoppers. This relationship was also recovered in previous phylogenetic efforts, including Bayesian analyses using more sophisticated CAT models (e.g., Bennett and Mao 2018). These results raise questions about the accuracy of phylogenetic inference within this clade and its potential inability to accurately reconstruct the origins and relationships of betaproteobacterial symbionts. Nevertheless, the relationships of *Vidania* lineages within Fulgoromorpha conform closely with our understanding of host family, genus, and species (fig. 5*B*; Song and Liang 2013; Urban and Cryan 2012).

## Discussion

Until recently, Fulgoromorpha has been largely overlooked in the field of endosymbiosis, despite their taxonomic diversity, ecological and economic significance, and diverse endosymbioses that are clearly distinct from those in the sister clade Cicadomorpha (Buchner 1965; Dietrich 2009). The newly sequenced genomes of *Sulcia* and *Vidania* from three *Pyrops* planthopper species expand our understanding of the evolutionary history in Fulgoromorpha. The genome of *Sulcia* and *Vidania* of three *Pyrops* planthoppers are highly similar to previously characterized strains, especially from the related Dictyopharidae family. The genome comparison of *Sulcia* lineages revealed that its genome organization has been highly conserved over its ∼300 Myr history of codiversification with the Auchenorrhyncha. Nevertheless, *Sulcia* still experienced genome inversions several times independently: once in the common ancestor of one of the two major auchenorrhynchan clades, and at least five times more recently (fig. 3). There is no doubt that *Sulcia* is derived from a single ancestral infection that occurred early in the evolution of the Auchenorrhyncha suborder (Moran et al. 2005). In contrast, however, the lack of genomic synteny among *Vidania*, *Nasuia*, and *Zinderia* beta-symbionts lineages raises doubts about whether they share a common origin.

### *Sulcia*, *Vidania*, and the Additional Microbial Symbionts in *Pyrops* Planthoppers

In *Pyrops* planthoppers, *Sulcia* synthesizes three EAAs and *Vidania* provides the remaining seven. The same pattern was observed in five previously investigated planthoppers from families Dictyopharidae and Cixiidae (Bennett and Mao 2018; Michalik et al. 2021; Gossett et al. 2023). This pattern is clearly distinct from that in Cicadomorpha, the sister clade of Fulgoromorpha, where *Sulcia* provides more amino acids than its companion (McCutcheon and Moran 2010; Bennett and Moran 2013). However, several pathways are incomplete and they lack genes responsible for one or more steps (e.g., arginine biosynthesis in *Vidania*; see fig. 2). These incomplete pathways may be rescued by the host, additional symbionts, moonlighting genes acquiring new functions, or by switching to alternative substrate usage for the nutrient production, as shown in other systems. For example, the loss of the ornithine biosynthesis pathway (*argABCDE*) in arginine biosynthesis, also observed in a few lineages of the nutritional endosymbiont *Buchnera* in aphids (Chong et al. 2019), is compensated by the host using proline rather than glutamate as the substrate for the ornithine synthesis, as suggested by proteomics (Poliakov et al. 2011). The gene losses in other pathways have been fully discussed by Bennett and Mao (2018).

The impressive symbiont diversity in planthoppers has been noted by Müller and Buchner in their classic microscopy work (Müller 1940, 1949; Buchner 1965). Recent studies combining modern microscopy techniques and molecular tools suggest that endosymbionts additional to *Sulcia* and *Vidania* may also play significant roles in nutrient provisioning and metabolite exchange (Bennett and Mao 2018; Michalik et al. 2021, 2023). For example, in the family Cixiidae, the gammaproteobacterium *Purcelliella* likely provides B vitamins and metabolic support for *Sulcia* and *Vidania* (Bennett and Mao 2018). In the family Dictyopharidae, the bacteria *Sodalis* and *Arsenophonus*, in the absence of *Purcelliella*, also encode genes involved in B vitamins synthesis (Michalik et al. 2021). The highly reduced *Sodalis*-like symbionts and the more recently acquired *Sodalis*/*Arsenophonus* symbionts in three *Pyrops* species may play similar roles. Their functions will be explored in a separate publication.

### Genome Rearrangements in *Sulcia*

The evolution of bacterial endosymbiont genomes is thought to be rapid and chaotic soon after they are acquired by an insect host (McCutcheon et al. 2019). Genes critical to the symbiosis are generally under strong selection and are maintained, while others experience relaxed selection and intense genetic drift (Moran 1996). In endosymbionts that follow strict vertical transmission, these redundant genes are eventually purged from the bacterial genomes (Bobay and Ochman 2017). The end result is usually a highly stable and compact bacterial genome, as shown in the ancient symbionts of diverse insects (e.g., *Buchnera* in aphids; Chong et al. 2019; *Blochmannia* in ants; Williams and Wernegreen 2015; *Blattabacterium* in cockroaches; Patiño-Navarrete et al. 2013; *Wigglesworthia* in tsetse flies; Rio et al. 2012). *Sulcia* is among the most stable symbionts identified so far (McCutcheon et al. 2009a; Bennett and Moran 2013). Indeed, although substantial differences in gene content and genome size exist between *Sulcia* lineages of Fulgoromorpha and Cicadomorpha, they retained a highly conserved gene order in their ∼300 Myr history of coevolution with insect hosts.

Despite the overall conserved gene order, several genome rearrangements have occurred in *Sulcia* during its codiversification with the Auchenorrhyncha. One took place during or soon after the divergence of Fulgoromorpha and Cicadomorpha, and others occurred much later in ancestors of different genera or tribes. These genome inversions were unexpected in a symbiont provided often as an example of genomic stability. However, occasional recombinations and rearrangements have occurred in other ancient endosymbionts of insects. In carpenter ants, the genome comparison between three divergent lineages of the nutritional endosymbiont *Blochmannia*, spanning ∼40 Myr, revealed eight inversions, consisting of two to 34 genes (Williams and Wernegreen 2015). In a more ancient symbiosis (∼140 Myr) in cockroaches, the comparison between five lineages of the primary endosymbiont *Blattabacterium* revealed three inversions, ranging between 2.9 and 242 kb (Patiño-Navarrete et al. 2013). In aphids, the genomic comparison of 39 strains of the obligate endosymbiont *Buchnera* revealed perfect synteny over 100 Myr, with the exception of a six-gene inversion shared by a few lineages (Chong et al. 2019). These rare genome structural changes reflect somewhat dynamic evolution in those highly stable and tiny genomes.

Genome inversions may be caused by an early wave of mobile elements seen in both recently acquired (e.g., *Serratia*; Manzano-Marín et al. 2012; *Sodalis*; Clayton et al. 2012) and ancient endosymbionts (e.g., *Portiera* in whiteflies; Sloan and Moran 2013). Then, these inversions become randomly fixed or purged by genetic drift. Alternatively, genome inversions may also be subject to natural selection. Inversions may bring fitness costs to bacteria by changing gene positions and creating replication-transcription conflicts that alter the gene expression level and mutation rate. These inversions may be negatively selected and weeded out of bacterial populations (Mackiewicz et al. 2001; Merrikh et al. 2012). On the other hand, inversions may also introduce structural polymorphism when both the ancestral and the inverted genomes exist. Such polymorphism has been observed in at least three endosymbionts, including *Portiera* in whiteflies (Sloan and Moran 2013), *Tremblaya* in mealybugs (McCutcheon and von Dohlen 2011), and *Hodgkinia* in cicadas (Łukasik et al. 2018). Such genomic structural variation could alter the expression pattern of genes involved in host-bacterial symbioses, providing evolutionary flexibility to adapt to environmental challenges (Hughes 2000; Bennett and Moran 2015; McCutcheon et al. 2019).

### Several Lines of Evidence Support the Independent Origin of *Vidania*

Like *Sulcia*, the coresident beta-symbionts in each Auchenorrhyncha superfamily have codiversified with their hosts for tens and maybe hundreds of millions of years (Moran et al. 2005; Bennett and Moran 2013; Johnson et al. 2018). Their genomes have gone through the dynamic phase of pseudogenization and degradation, becoming tiny, compact, and stable. That stability is evident in a comparison between *Nasuia* strains separated by >100 Myr, or *Vidania* strains separated by ∼200 Myr (fig. 4; Johnson et al. 2018). It may be expected that beta-symbionts should share similar patterns of synteny if they descended from a single common ancestor as *Sulcia* did, and as has been observed in other ancient insect symbionts (e.g., Buchnera; Chong et al. 2019). However, this is not what we found when comparing the genomes of the three genera (fig. 4). Remarkably, *Vidania*, *Nasuia*, and *Zinderia* have little to no synteny or chunks of shared gene order. Given the general patterns for symbiont lineages with tiny genomes, this result provides a strong argument that the three beta-symbiont lineages may not be derived from a common ancestral infection. On the contrary, these patterns support the scenario where each of the major auchenorrhynchan host lineages that harbor a distinct beta-symbiont lineage (e.g., *Zinderia* in spittlebugs and *Nasuia* in leafhoppers) acquired novel symbionts independently, early in their diversification.

Despite expectations of genomic synteny in ancient symbionts, other evolutionary processes could also result in significant changes in the genome structure of closely related lineages (Sloan and Moran 2013; Santos-Garcia et al. 2020). Specifically, in some clades of whiteflies, their ancient nutritional endosymbiont *Portiera* accumulated repetitive sequences and expanded intergenic regions, causing extensive recombinations and rearrangements in the genome. Interestingly, syntenically expanded genomes were observed in three host lineages separated by at least 7 Myr, indicating a return to the relatively stable stage after the period of instability (Santos-Garcia et al. 2020). We could envision that a similar genome expansion occurred early in the evolution of beta-symbionts among the major Auchenorrhyncha lineages. However, in the case of *Portiera*, the genome expansion was linked to the loss of the DNA polymerase proofreading subunit (*dnaQ*), which is still retained by all examined lineages of *Vidania*, *Nasuia*, and *Zinderia*. Nevertheless, these beta-symbionts lost the DNA mismatch repair system (*mutS* and *mutL*) that has a strong stabilizing effect on the maintenance of genome structure (Nilsson et al. 2005), which possibly contributed to the genome instability in the early divergence of beta-symbionts.

We expected that the taxonomically improved phylogeny, including significantly more beta-symbiont genomes, could further resolve the pending issue of beta-symbiont origin. However, the new phylogeny is largely in agreement with previous efforts (Bennett and Moran 2013; Koga et al. 2013; Bennett and Mao 2018), producing a highly supported clade of the three beta-symbionts. Unfortunately, phylogenetic analyses on those rapidly evolving and extremely reduced genomes all face inevitable phylogenetic errors coming from the taxonomic sampling limitations, extremely elevated rates of molecular evolution, and strong AT nucleotide bias that increase the potential for spurious results like long-branch attraction (Bennett and Mao 2018). These errors are evident in our analysis as *Tremblaya and Profftella*, the endosymbiont of distantly related scale insects and psyllids, were placed within the same clade as Auchenorrhynchan beta-symbionts. In addition, the relationship between *Vidania*, *Nasuia*, and *Zinderia* in the phylogeny do not agree with the host phylogeny (Cryan and Urban 2012; Skinner et al. 2020). Reconstructing the evolutionary origin of these major beta-symbiont lineages from genomes that have been reduced to <5% of the size of most free-living ancestors is a persistent challenge. It raises the question of whether phylogenetic tools are capable of doing it at all. Hence, we explored whether other evidence (e.g., genomics, metabolisms, bacteriome organization, symbiont cell histology) could support either side of the argument for the origin of beta-symbionts (table 1).

**Table 1:** The Comparison of Biological Characteristics Between Beta-Symbionts

Category	Similarities and dissimilarities between beta-symbionts	References
Genomics	Lack of synteny among *Vidania*, *Nasuia*, and *Zinderia*	This study
	UGA stopped codon reassignment to Trp in *Nasuia* and *Zinderia*, but not in *Vidania*	McCutcheon and Moran 2010; Bennett and Moran 2013; Bennett and Mao 2018;
Metabolism	*Nasuia* and *Zinderia* encode the direct sulfhydrylation pathway (*MetX*) in methionine biosynthesis. However, they use two different sulfhydrylases, as succinyl-homoserine sulfhydrylase (*MetB*) in *Nasuia* and acetyl-homoserine sulfhydrylase (*MetY*) in *Zinderia*. *Vidania* uses the transsulfuration pathway (*metABC*) instead	McCutcheon and Moran 2010; Bennett and Moran 2013; Bennett and Mao 2018; Michalik et al. 2021; This study
	*Vidania* produces seven amino acids, while *Zinderia* produces three and *Nasuia* produces two	
Phylogenetics	*Vidania*, *Nasuia*, and *Zinderia* were grouped in a highly supported clade	Bennett and Moran 2013; Koga et al. 2013; Bennett and Mao 2018; Cryan and Urban 2012; Skinner et al. 2020; This study
	The basal position of *Zinderia* among beta-symbionts in the phylogeny does not agree with the host phylogeny	
Bacteriome organization	*Vidania* occupies a separate bacteriome from *Sulcia* while *Nasuia* and *Zinderia* share the same bacteriome with *Sulcia*	McCutcheon and Moran 2010; Bennett and Moran 2013
Morphology	*Vidania* has a strikingly large and often irregularly lobed shape in mature bacteriome, while *Nasuia* and *Zinderia* are often regularly shaped.	Buchner 1965; Bressan and Mulligan 2013; Kobiałka et al. 2018; Michalik et al. 2021
Transmission	Both *Vidania*, *Nasuia*, and *Zinderia* share the same transovarial transmission strategy	Szklarzewicz and Michalik 2017; Huang et al. 2020; Michalik et al. 2021

The reassignment of the UGA stop codon to tryptophan in both *Nasuia* and *Zinderia* (McCutcheon and Moran 2010; Bennett and Moran 2013), rare in other reduced endosymbiont genomes (Bennett and Moran 2013), was considered as a strong argument for the close relationship between *Nasuia* and *Zinderia*. They also share the same loss of the peptide chain release factor 2 (*prfB*) that recognizes the UGA stop codon. In contrast, *Vidania* retains the *prfB* gene and does not use the alternative code (Bennett and Mao 2018). This finding seems to contradict the single ancestry of *Vidania* and *Nasuia*/*Zinderia*. However, the patterns could be explained by the loss of *prfB* gene in the common ancestor of *Nasuia* and *Zinderia* after its divergence from *Vidania*. Notably, *Hodgkinia*, the independently acquired alphaproteobacterial symbiont in the Cicadoidea superfamily, also lost *prfB* and uses an alternative genetic code (McCutcheon et al. 2009a, 2009b). The finding that genetic code reassignment can occur convergently in this group of symbionts of auchenorrhynchan insects weakens argumentation about whether this feature should be given much weight in relationship reconstructions.

More insights into symbiont origins come from the comparisons of how biosynthesis of the ten EAAs is partitioned among beta-symbionts and *Sulcia*. In Cicadomorpha, *Sulcia* plays a major role. In spittlebugs and leafhoppers, *Sulcia* provides seven and eight amino acids, respectively, while *Zinderia* and *Nasuia* provide the remaining three and two (McCutcheon and Moran 2010; Bennett and Moran 2013). Strikingly, this role is reversed in Fulgoromorpha where *Sulcia* provides only three amino acids while *Vidania* provides seven (Bennett and Mao 2018; Michalik et al. 2021). These patterns are again suggestive of independent origins of Fulgoromorpha and Cicadomorpha symbionts but are not conclusive. The previously characterized multisymbiont complexes in sap-feeding Hemiptera showed a striking degree of functional complementarity (Husnik and McCutcheon 2016), indicating that the loss of redundant copies of essential genes generally happens quickly, but stochastic processes could lead to differential gene loss among symbionts in different host lineages originating from a single ancestor. One can imagine that the divergence of Cicadomorpha and Fulgoromorpha occurred soon after the acquisition of *Sulcia* and the beta-symbionts, during which the biosynthetic pathways were lost in a differential manner. On the other hand, however, it would have been surprising if the tryptophan biosynthesis pathway, the one retained by *Zinderia* but not *Nasuia*, was then retained for additional tens of millions of years until the divergence of leafhoppers and spittlebugs.

The details of the methionine biosynthesis pathway also vary among three beta-symbionts. *Nasuia* and *Zinderia* use direct sulfhydrylation pathway (*MetX*) in the production of methionine, but two different sulfhydrylases are used: succinyl-homoserine sulfhydrylase (*MetB*) in *Nasuia* and acetyl-homoserine sulfhydrylase (*MetY*) in *Zinderia* (Bennett and Moran 2013). In contrast, *Vidania* uses the transsulfuration pathway (*metABC*). The differences in methionine synthesis pathways further point to independent origins for the Fulgoromorpha and Cicadomorpha beta-symbionts, further suggesting that *Nasuia* and *Zinderia* are also derived from different original infections. Nevertheless, the different methionine pathways could also be explained by horizontal gene transfer (Gophna et al. 2005), or a common ancestor having multiple gene copies, one of which was differentially lost in the major host lineages (Bennett and Moran 2013).

The internal organization of the host bacteriome organ may also shed light on beta-symbiont origins. Beta-symbionts in two major Auchenorrhyncha clades differ significantly in their bacteriome organization. In Cicadomorpha, *Sulcia* and beta-symbionts colonize different regions of a single bacteriome (Buchner 1965; Noda et al. 2012; Koga et al. 2013; Łukasik et al. 2018). This contrasts with Fulgoromorpha, where each endosymbiont is confined to its own, physically distinct bacteriome organ (Buchner 1965; Bressan and Mulligan 2013; Michalik et al. 2021). The difference in bacteriome organization between the major lineages suggests that symbionts in the two clades have independent origins, infecting different Fulgoromorpha and Cicadomorpha host tissues that are later established as bacteriome organs. Nevertheless, the difference in bacteriome organization could also simply reflect the evolution of codependency among symbiont species. Symbionts living in adjacent bacteriocytes are more likely to exchange metabolites directly, which has been proposed in Cicadomorpha (McCutcheon and Moran 2007; Douglas 2016). This intimacy could also be seen in Fulgoromorpha, as in the family Cixiidae where the third symbiont *Purcelliella*, often sharing the same bacteriome with *Vidania*, may provide metabolites for completing the methionine synthesis pathway (Bressan and Mulligan 2013; Bennett and Mao 2018).

Finally, *Vidania* is known for its unusual morphology: strikingly large and often irregularly lobed cells in the mature bacteriome, clearly different from rod-like and regularly shaped cells of *Nasuia* and *Zinderia* (Buchner 1965; Bressan and Mulligan 2013; Kobiałka et al. 2018; Michalik et al. 2021). Interestingly, *Vidania* has a second morphotype, represented by round and regularly shaped cells, in the rectal organ that is unique to female planthoppers and absent in leafhoppers or spittlebugs. This second morphotype, resembling cells undergoing transovarial transmission, was proposed as an infectious form ready to be transmitted to the progeny (Bressan and Mulligan 2013; Michalik et al. 2021). The mechanisms underlying the morphological changes of *Vidania* are still unclear. Nevertheless, morphology is yet another clear difference between Fulgoromorpha and Cicadomorpha symbionts, supporting their independent origins.

## Conclusion

There is growing evidence that the tremendous diversity of symbioses of sap-feeding Hemipterans, and many other insects, was shaped by independent, repeated colonization events, and often serial replacements, by different microbes. These microbes include versatile opportunists similar to *Sodalis praecaptivus* (Clayton et al. 2012; McCutcheon et al. 2019), or specialized pathogens like *Ophiocordyceps* fungi (Matsuura et al. 2018). Following colonization, they undergo dynamic genomic reduction in parallel, sometimes in a convergent manner, complementing the function of other symbionts present in the host (Husnik and McCutcheon 2016). Unfortunately, due to limited sampling and challenges with phylogenetic reconstructions, we are still far from understanding the nature, dynamics, and evolutionary consequences of these replacements, even for relatively recent symbioses. For those that are hundreds of millions of years old, our standard comparative phylogenetic and phylogenomic tools may not be able to accurately and conclusively reconstruct their histories. The combination of a holistic biological approach with more thorough taxonomic sampling across early divergences will give us the best chance of accurately reconstructing the origin and history of these complex symbioses.

Among the Auchenorrhyncha, which is an emergent model for ancient complex symbioses, there is still uncertainty regarding the origins of symbioses in these insects. With the inclusion of more genomic resources for *Vidania* in a diversity of planthopper species, it has become clear that few to no genomic, metabolic, and morphological traits suggest that this symbiont is derived from a common ancestor shared with the other betaproteobacterial symbionts, *Nasuia* and *Zinderia*, from leafhoppers and spittlebugs, respectively. Instead, we hypothesize that *Vidania* was derived from an independent origin, which could help to explain its dramatically different nutritional responsibilities and other traits relative to *Nasuia* and *Zinderia* (Michalik et al. 2021). It is also worth noting that the alphaproteobacterial partner symbiont of *Sulcia* in cicadas, *Hodgkinia*, also has extremely reduced genomes, encodes two amino acid biosynthetic pathways, uses an alternative genetic code, inhabits the same bacteriome as Sulcia, and shares the same transovarial transmission strategy (McCutcheon et al. 2009a, 2009b; Szklarzewicz and Michalik 2017; Huang et al. 2020; Michalik et al. 2021). With the exception of the methionine biosynthesis strategy, *Hodgkinia* is about as similar to *Nasuia* and *Zinderia* as these lineages are to each other, making it clear the similarities among symbionts can arise through convergent evolution as well as through shared origins. Nevertheless, both hypotheses of independent acquisition of different symbionts by the ancestors of at least the Cicadomorpha and Fulgoromorpha, or a single infection in the ancestor to the auchenorrhyncha, remain viable hypotheses. The latter suggests extensive varying evolutionary pressures and constraints that led to differences in genome organization and function. In the future, approaches such as transcriptomics and proteomics, and the comparison of host support mechanisms between species harboring divergent microbes, may resolve the question about the origin of these symbioses, while helping understand their evolution and function (Mao et al. 2018; Mao and Bennett 2020).

## Materials and Methods

### Sample Processing and Metagenomic Sequencing

Individuals of three *Pyrops* species (*Pyrops lathburii* [PYRLAN], *P. clavatus* [PYRCLA], and *P. viridirostris* [PYRVIR]) were sampled from their natural habitat in Vietnam in 2019. Bacteriomes were dissected from a single female for each species. We extracted High-Molecular Weight Genomic DNA (HMW DNA) from the dissected bacteriomes with MagAttract HMW DNA Kit (QIAGEN). Metagenomic libraries of the three species were prepared with NEBNext Ultra II DNA Library Prep Kit (BioLabs, New England) with a target insert length of 350 bp and sequenced on Illumina NovaSeq 6000 S4 (2 × 150 bp reads). For long-read sequencing, libraries were prepared with Ligation Sequencing Kit (SQK-LSK 109), and sequenced on MinION Mk1C (Oxford Nanopore Technologies) with R9.4.1 Flow Cell.

### Symbiont Diversity in Bacteriome Samples

Initially, we used phyloFlash v3.3 (Gruber-Vodicka et al. 2020) to determine the diversity of symbionts by reconstructing the sequences of small subunit ribosomal RNAs (SSU rRNAs; 16S and 18S rRNAs). We then conducted metagenomic assembly (described in the next section) and used anvi-get-sequences-for-hmm-hits from the anvi’o platform (Eren et al. 2015) to identify host and symbiont rRNA gene-containing contigs in the Illumina assembly. rRNA sequences were blasted against the NCBI database to determine their taxonomies. We also used NanoTax.py (https://github.com/diecasfranco/Nanotax) to classify contigs into taxonomic groups determined in the previous step. NanoTax.py performs blast searches using assembled contigs against a customized nucleotide database and a protein database containing sequences from previously assembled genomes of hosts, symbionts, and their free-living relatives. Other information, including the GC content, coverage, and length of each contig, is compiled into output with the assigned taxonomy. Contigs identified as representing symbionts and larger than 1500 base-pairs (bp) were plotted with Processing 3 (http://www.processing.org) in figure 1*B*.

### Symbiont Genome Assembly

We conducted metagenomic assemblies as follows: 1) read quality filtering; 2) draft genomes assembly with Nanopore and Illumina reads separately; 3) symbiont genome identification from Nanopore assembly; 4) genome polishing with Nanopore and Illumina reads; and, 5) final genome quality check.

Initially, NanoFilt v2.7.1 (settings: -l 500 –headcrop 10 -q 10) was used to extract high-quality Nanopore reads (De Coster et al. 2018). Similarly, trim_galore v0.6.4 (settings: –length 80 -q 30; https://github.com/FelixKrueger/TrimGalore) was used to trim the adapter and control the quality of Illumina paired-end reads. The quality of filtered reads was checked using FastQC v0.11.9 (https://github.com/s-andrews/FastQC). In the second step, Canu v2.1.1 was used to assemble metagenomes from high-quality Nanopore reads (settings: –genome-size 5 month and other default settings; (Koren et al. 2017)). Illumina reads were assembled using MEGAHIT v1.1.3 with k-mer size from 99 to 255 (Li et al. 2016). In the third step, contigs of *Sulcia*, *Vidania*, and other microbial symbionts were identified using blastn and blastx against custom databases, which included DNA and amino acid sequences of reference genomes of hemipteran insects, mitochondria, microbial symbionts, and their free-living relatives. *Sulcia* and *Vidania* draft genomes, all confirmed circular by Canu were processed further through the polishing step. In the fourth step, high-quality Nanopore reads were trimmed and split using a custom script NanoSplit.py (https://github.com/junchen-deng/NanoSplit), which detects and removes low-quality regions with a sliding-window. All draft genomes were polished by medaka v1.2.1 (https://github.com/nanoporetech/medaka) with the model r941_min_high_g360 for one round and by Pilon v1.23 (Walker et al. 2014) using Illumina reads for at least two rounds to fix any mismatches, insertions, and deletions.

In the final step, the polished genomes’ quality was checked by mapping reads to each genome. The original Nanopore reads were filtered by NanoFilt v2.7.1 to recover reads with lengths longer than 1500 bp and an average read quality score >10. Filtered Nanopore reads was then mapped onto polished genomes using minimap2 v2.17 (r941) (Li 2018). Similarly, the paired-end Illumina reads were mapped onto each genome using bowtie2 v2.4.2 (Langmead and Salzberg 2012). All mappings were visualized on Tablet (Milne et al. 2013) to verify even and consistent coverage (e.g., no breaks).

### Genome Annotation

The genomes of *Sulcia* and *Vidania* were annotated with a custom Python script modified from (Łukasik et al. 2018). The script first extracted all the Open Reading Frames (ORFs) and their amino acid sequences from each genome. These ORFs were searched recursively using HMMER v3.3.1 (Eddy 2011) against custom databases containing manually curated sets of protein-coding, rRNA, and noncoding RNA (ncRNA) genes from previously characterized *Sulcia* or *Vidania* lineages. rRNA and ncRNA genes were searched with nhmmer (HMMER V3.3.1) (Wheeler and Eddy 2013), and tRNAs were identified with tRNAscan-SE v2.0.7 (Chan et al. 2021). Based on the relative length compared to the reference genes, protein-coding genes were classified as functional (>85%), putative pseudogenes (>60%), or pseudogenes (<60%). Any ORFs over 300 bp but with no significant similarity to any reference genes were blasted against UniProt (The UniProt Consortium et al. 2021) and NCBI databases and compared carefully to the top hits. Genes without any annotations were marked as “hypothetical”. All biosynthesis pathways of EAAs were manually constructed with MetaCyc database (Caspi et al. 2020).

### Genome Comparisons

For the gene set comparison among *Sulcia* and *Vidania* lineages (fig. 1*C*), we selected major functional groups that included amino acid biosynthesis, protein folding and stability, replication and repair, transcription, translation, ribosome-related, RNA-related, aminoacyl tRNA synthetases, ribosomal subunit protein, and TCA cycle. Hypothetical genes and genes not involved in any of the above functional groups were not included in the comparison. The genome synteny comparison among *Sulcia* and beta-symbionts (*Vidania*, *Nasuia*, and *Zinderia*) included all published lineages with a recognizable taxonomy available on GenBank (supplementary material table S1, Supplementary Material online). The genome synteny comparison among all lineages was illustrated with PROmer v3.07 and MUMmerplot v3.5 (Kurtz et al. 2004). Other figures were produced by Processing 3 and were edited in Inkscape.

### Host and Symbiont Phylogenetics

To interpret the results of genome comparison with the host phylogeny, we reconstructed the maximum likelihood phylogeny of host insects based on the concatenated set of ten mitochondrial genes (*nad2*, *cox1*, *cox2*, *atp6*, *cox3*, *nad3*, *nad6*, *cob*, *nad1*, and *rrnL*). The complete mitochondrial genomes of three *Pyrops* planthoppers were assembled and annotated following the methods described in the previous sections. The mitochondrial markers from other host species were extracted from previously published mitochondrial genomes (supplementary material table S1, Supplementary Material online). Note that the mitochondrial genomes of *Oliarus filicicola* (OLIH) and *Clastoptera arizonana* (CARI) were unavailable on NCBI, and we replaced them with related species, *Oliarus cf. filicicola* HI01081 and *Philaenus spumarius*, in the analysis. Accordingly, the tree branches of OLIH and CARI were dotted in figures 1 and 3–4. The phylogenetic analyses were conducted in IQ-Tree on XSEDE (Minh et al. 2020) and implemented in CIPRES v.3.3 (Miller et al. 2010). “Model Selection” (Kalyaanamoorthy et al. 2017) was selected to search for the best model in CIPRES. The partition type was set to allow the ten partitions (one for each marker) to have different speeds (Chernomor et al. 2016). “TESTNEWMERGE” was specified to allow partitions with similar speeds to be analyzed as a single partition. The best-fit models were decided by the highest BIC (Bayesian information criterion) scores. Bootstrapping was conducted using “SH-aLRT” bootstrap (BS) methods with 1,000 replicates. All other setting options were set as default.

To determine the relationships of the sequenced betaproteobacterial strains, as well as their placement in the larger betaproteobacterial phylogeny, we reconstructed their relationships using a core-set of protein-coding genes and the 16S + 23S rRNA cassette. In addition to previously published *Vidania*, *Nasuia*, and *Zinderia* genomes, we extracted gene sequences from unpublished, draft *Vidania* genomes from 23 planthopper species from 12 families (supplementary material table S4, Supplementary Material online). Orthologs were determined using HMMER3 searches in Phyloskeleton v.1.1.1 against the 109 bacterial panortholog gene set (settings: *e*-value = 0.01, best-match-only; Darriba et al. 2011; Eddy 2011; Guy 2017). Genes were translated to amino acid sequences and aligned with Mafft v7 (settings: L-INS-I model; Katoh and Standley 2013). Models of amino acid substitution were determined with Prottest3 and ambiguously aligned regions trimmed with Trimal v1.4 (Capella-Gutierrez et al. 2009; Darriba et al. 2011; supplementary material Data S1–2, Supplementary Material online). Trimmed gene alignments were concatenated into a matrix of 289 taxa and 31 genes (7,240 amino acid sites) for phylogenetic analysis (supplementary material tables S2–3, Supplementary Material online).

For comparison, an expanded 16S and 23S data set were built from the entire NCBI RefSeq complete genome database for *Betaproteobacteria* (accessed April 13, 2023; O’Leary et al. 2016). The RefSeq dataset was supplemented with symbionts mentioned above and additional strains of *Tremblaya*, *Profftella*, as well as several strain of “*Candidatus* Dactylopiibacterium carminicum” (supplementary material table S5, Supplementary Material online). The extracted rRNA genes were individually aligned using Mafft v7 (settings: E-INS-i model for large alignments with multiple domains; Katoh and Standley 2013). Alignments were subsequently checked for unalignable, misidentified, or missing genes. Individual rRNA alignments were then concatenated into a matrix of 382 taxa and 6,011 nucleotide sites (supplementary material Data S5, Supplementary Material online).

RAxML v.8 was used to infer Maximum Likelihood phylogenies from concatenated alignments that were partitioned by gene and run for 500 bootstrap replicates (Stamatakis 2014). Two parallel analyses were run for amino acid matrix (-m PTROCATLG) and a Dayhoff6 recoded matrix (-m MULTIGAMMA -K GTR) in an effort to reduce phylogenetic artifacts (Dayhoff et al. 1978). For the rRNA two-gene matrix, an ML phylogeny was inferred from a gene partitioned concatenated alignment (-m GTRCAT) run for 500 bootstrap replicates.

## Supplementary Material

evad120_Supplementary_Data

## Data Availability

The genomes of *Sulcia* and *Vidania* from three *Pyrops* planthoppers are available under the BioProject PRJNA821037, PRJNA821196, and PRJNA821197 in NCBI databases. The host mitochondrial genomes are available under the accession numbers ON209295-ON209297 in GenBank. Other data underlying phylogenetic analyses (supplementary material Data Files S1–6, Supplementary Material online) are available in the GitHub repository (https://github.com/junchen-deng/Supplementary-Material_DengEtal_pyrops_2022).
